# *AR* Expression Correlates with Distinctive Clinicopathological and Genomic Features in Breast Cancer Regardless of *ESR1* Expression Status

**DOI:** 10.3390/ijms231911468

**Published:** 2022-09-29

**Authors:** Mengping Long, Chong You, Qianqian Song, Lina X. J. Hu, Zhaorong Guo, Qian Yao, Wei Hou, Wei Sun, Baosheng Liang, Xiaohua Zhou, Yiqiang Liu, Taobo Hu

**Affiliations:** 1 Department of Pathology, Peking University Cancer Hospital, Beijing 100083, China; 2 Chongqing Research Institute of Big Data, Peking University, Chongqing 401121, China; 3 Department of Biostatistics, School of Public Health, Peking University, Beijing 100191, China; 4 Department of Pathology, Alaska Native Medical Center, Anchorage, AK 99501, USA; 5 Department of Breast Center, Peking University Cancer Hospital, Beijing 100083, China; 6 Department of Breast Surgery, Peking University People’s Hospital, Beijing 100044, China

**Keywords:** androgen receptor, estrogen receptor, breast cancer, molecular feature

## Abstract

Androgen receptor (AR) expression is frequently observed in breast cancer, but its association with estrogen receptor (ER) expression in breast cancer remains unclear. This study analyzed the clinicopathological and molecular features associated with AR negativity in both ER-positive and ER-negative breast cancer, trying to elucidate the molecular correlation between AR and ER. Our results showed that AR negativity was associated with different clinicopathological characteristics and molecular features in ER-positive and ER-negative breast cancer. Moreover, AR-positive breast cancer has better clinicopathological features than AR-negative breast cancer, especially in the ER-negative subtype. These results suggest that the role of AR in ER-negative breast cancer is distinctive from that in ER-positive breast cancer.

## 1. Introduction

Breast cancer was the most common malignancy in women, of which 70–80% of cases expressed steroid hormone receptors, including estrogen receptor (ER) and progesterone receptor (PR) [1,2]. ER-positive breast cancer was estrogen-dependent and was primarily driven by the activated ER pathway, which was also effectively used as a therapeutic target. As another hormonal receptor, the androgen receptor (AR) was expressed in 70–85% of all breast cancer cases [3], and that ratio was about 10–63% in triple-negative breast cancer (TNBC), which did not express ER, PR, or HER2 [4,5,6]. On the other hand, for ER-positive breast cancer, AR was expressed in 70–95% of cases, varying in different studies [5,7]. The expression of AR was related to a good prognosis in early breast cancer in terms of both disease-free survival and overall survival [8,9]. Moreover, in ER-positive and ER-negative cancer, the expression of AR was reported to have opposite prognostic values as AR expression was correlated with increased DFS in luminal breast cancer and decreased DFS in triple-negative breast cancer (TNBC) [10].

While AR expression was more prevalent in breast cancer than ER [11,12], the detailed molecular role of AR in breast cancer remains unresolved. AR has previously been shown to support estradiol-mediated ER activity in ER-positive/AR-positive breast cancer [13,14]. AR inhibition can be synergized with tamoxifen to reduce the proliferation of ER-positive breast cancer [13]. A recent study showed that AR act as a tumor suppressor in ER-positive breast cancer by inhibiting the binding of ER to the estrogen response elements (EREs) and consequently suppressing the activated ER pathway [15]. Moreover, the *AR* pathway’s activity calculated by the gene panel expression was positively correlated with disease-free survival in ER-positive breast cancer, suggesting that AR expression is a protective factor in ER-positive breast cancer [16]. Therefore, an AR agonist instead of an antagonist should be applied in ER-positive breast cancer. However, studies showed that in ER-negative breast cancer, the expression of *AR* activates subsequent transcriptional programs, and its activation can promote cell proliferation [17,18]. Although the prognostic value of AR expression in ER-negative breast cancer was controversial, the AR antagonist enzalutamide has been investigated for the treatment of TNBC patients with a clinical benefit rate (CBR) reaching 33% at 16 weeks [19]. Selective androgen receptor modulators (SARMs) are clinically available and are being investigated as medicine for AR-positive breast cancer [20,21]. It is essential to understand the role of AR in ER-positive and ER-negative breast cancer for the precise application of SARMs in breast cancer.

In this study, we analyzed the clinicopathological and molecular features associated with AR negativity in both ER-positive and ER-negative breast cancer. We excluded HER2-amplified cases to elucidate the molecular correlation between AR and ER. Our results show that AR-positive breast cancer has better clinicopathological features than AR-negative breast cancer, especially in the ER-negative subtype.

## 2. Results

### 2.1. AR Expression and Clinicopathological Features in ER-Positive and ER-Negative Breast Cancer

**Figure 1:**
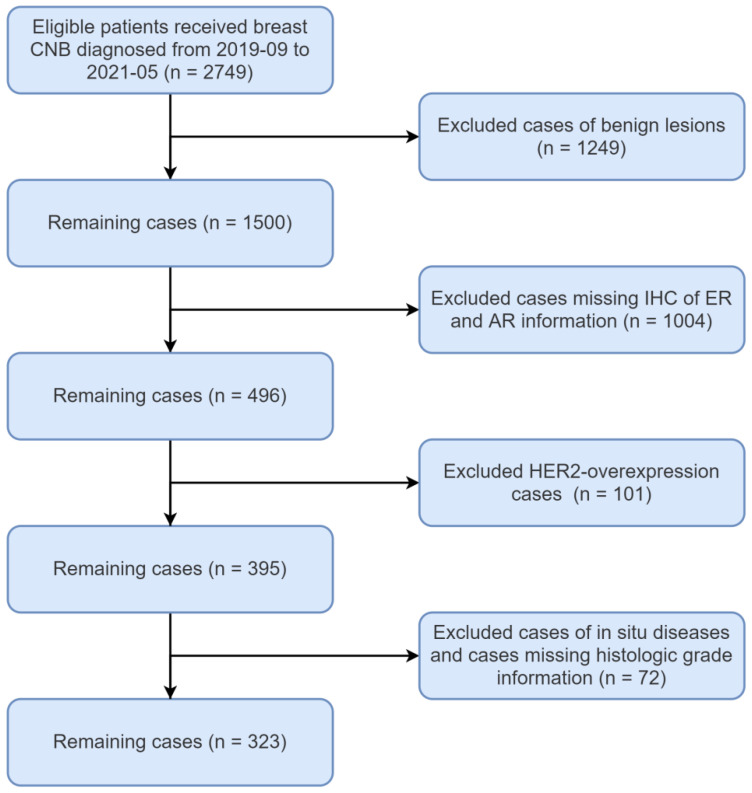
Flow chart of patient selection form the Peking University Cancer Hospital (PKUCH) cohort.

A total of 323 invasive breast cancer cases were recruited in our study, diagnosed from September 2019 to May 2021 in Peking University Cancer Hospital (Figure 1). All cases have immunostaining or fluorescent in situ hybridization (FISH) results of ER, PR, AR and HER2. Positivity of ER, PR and AR was defined as ≥1% of cancer cells showing positive nuclear staining. Two hundred and seventy-four cases (85%) were ER-positive and forty-nine (15%) cases were ER-negative. The clinicopathological characteristics between the AR+ and AR− groups were analyzed and compared in ER-positive and ER-negative breast cancer, respectively (Table 1 and Table 2).

**Figure 2:**
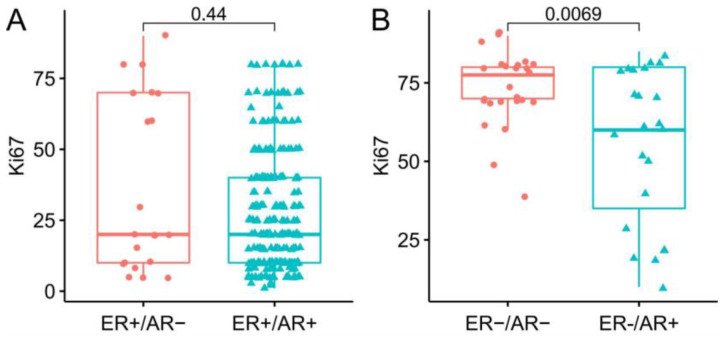
Comparison of Ki67 expression between AR+ and AR− groups. Box plot of Ki67 proliferation index in ER+ breast cancer (**A**) and in ER− breast cancer (**B**), respectively, in the PKUCH cohort.

In ER-positive breast cancer, PR expression was the only clinicopathological characteristic that exhibited a significant difference between ER+/AR− and ER+/AR+ groups. The expression of PR was significantly lower in the ER+/AR− group than in the ER+/AR+ groups (*p* = 0.01), indicating a common mechanism behind the negativity of expression for the two steroid hormone receptors (Table 1). In the ER-negative breast cancer group, the expression of PR also showed the same trend, although the *P* value was marginal (*p* = 0.09). Patients diagnosed with ER−/AR+ breast cancer were 9 years older than those with ER−/AR− breast cancer, which is consistent with previous findings [4]. The histologic grade of the ER−/AR+ and ER−/AR− groups showed a significant difference with AR negativity correlated with a more advanced histologic grade. Nevertheless, the positivity of CK5/6 and EGFR, which can serve as an indicator for basal-like character [22,23], was not statistically different between the ER−/AR+ and ER−/AR− groups (Table 2). The above results indicated that AR-negativity in ER-negative breast cancer has a stronger impact than AR-negativity in ER-positive breast cancer. To further confirm this, the Ki67 proliferation index was plotted and compared (Figure 2). No difference was detected in ER-positive breast cancer while the Ki67 index was much higher in the ER−/AR− group than in the ER−/AR+ group.

### 2.2. Estrogen Receptor Expression Correlates with Androgen Receptor Expression

**Figure 3:**
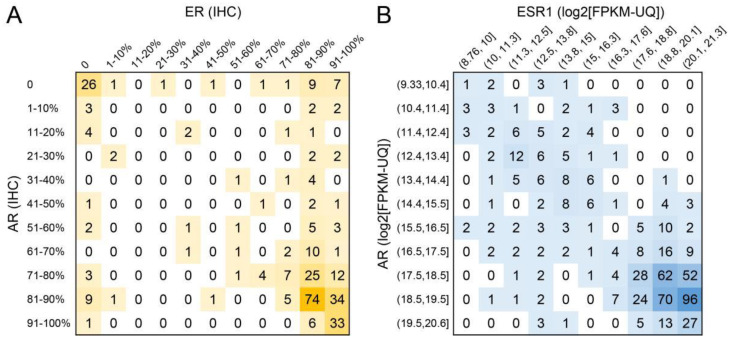
Summarization of the number of patients with specific AR and ER expressions. (**A**) Cell percentage categories of AR and ER expression in IHC from the PKUCH cohort. The case number of each specific AR and ER expression status were summarized and presented. AR and ER expression were reported either as negative or as the percentage of cells that showed positive expression which was sub-grouped into 10 categories with a 10 percent interval. (**B**) The expression profile of *AR* and *ESR1* in HER2- breast cancers from TCGA database. The expression of *AR* and *ESR1* genes were divided into ten equal parts according to the maximum and minimum value and presented in the heatmap.

**Figure 4:**
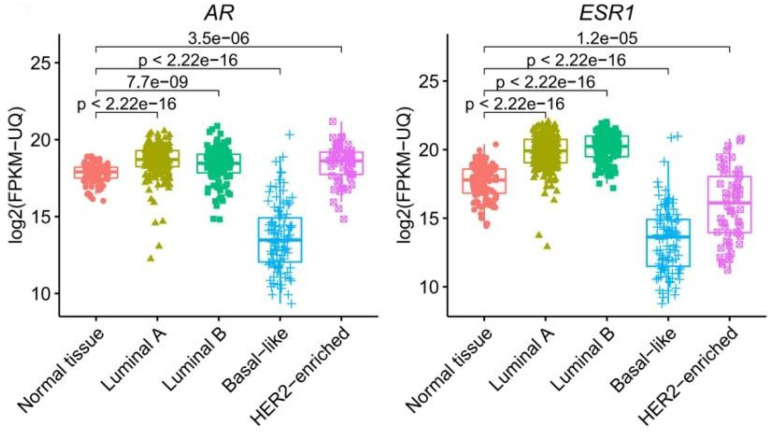
Expression of *AR* and *ESR1* gene in normal breast tissue and four intrinsic subtypes of breast cancer in TCGA cohort.

To further examine the correlation between ER and AR, their expression measured by IHC was plotted in Figure 3A. At the IHC level, it can be noticed that though the expression of AR was mostly enriched in ER-high expression cases, there were many cases in which the expression of AR and ER were discordant. Additionally, we have selected 466 HER2-negative breast cancer cases in The Cancer Genome Atlas (TCGA) database. The expression of *AR* and *ESR1* genes were displayed as a heatmap in Figure 3B. The expression of *AR* and *ESR1* were highly correlated. To further examine the correlation of AR and ER in different subtypes of breast cancer, the expression of *AR* and *ESR1* in the five intrinsic subtypes of breast cancer defined by PAM50 along with those in normal breast tissue was plotted in Figure 4.

**Figure 5:**
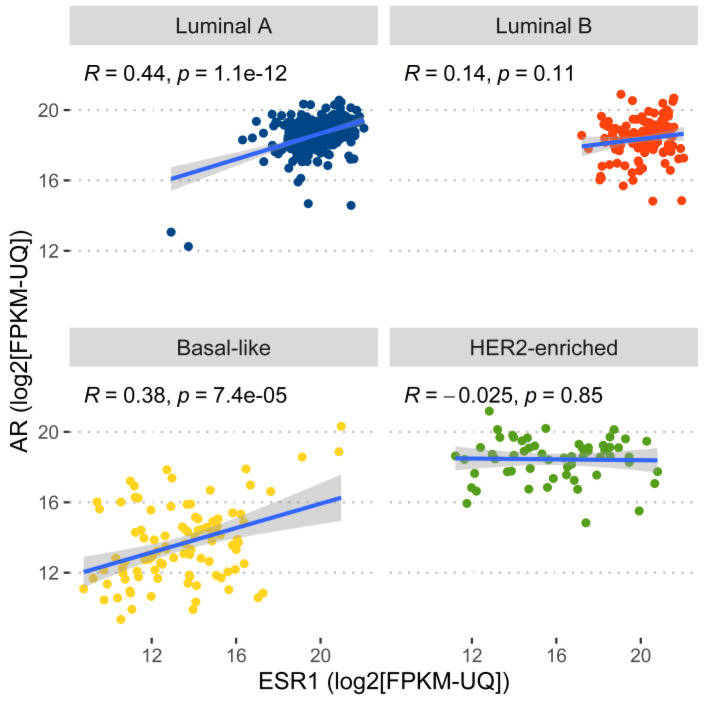
Correlation plot of the expression of *AR* and *ESR1* in four intrinsic subtypes of breast cancer. The regression line, Pearson’s correlation coefficient, and *p*-value were also displayed.

Compared to normal breast tissue, the expressions of *AR* and *ESR1* were higher in luminal A and B subtypes and lower in the basal-like subtype. However, in the HER2-enriched subtype, *AR* exhibited higher expression compared to normal breast tissue while *ESR1* was expressed at a lower level than normal breast tissue. The correlation between the expression of *AR* and *ESR1* was only significant in the luminal A and basal-like subtype while not significant in the luminal B and HER2-enriched subtype (Figure 5).

### 2.3. Androgen Receptor Expression Regulation Was Different in ER-Positive and ER-Negative Breast Cancer

**Figure 6:**
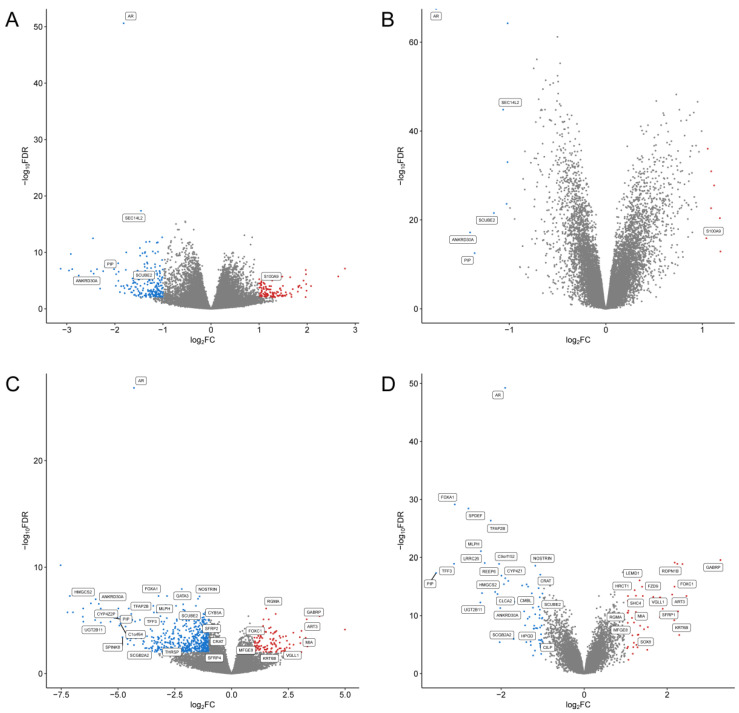
DEGs between AR-high and AR-low groups. in ER+ and ER− breast cancer. In ER+ breast cancer, DEGs between the AR-low and AR-high groups were identified and displayed using TCGA (**A**) and METABRIC (**B**) datasets, respectively. In ER− breast cancer, the DEGs between the AR-low and AR-high groups are identified in TCGA (**C**) and METABRIC (**D**) datasets, respectively. Dots in red color denote genes that are up-regulated in AR-low group while dots in blue denotes down-regulated genes in AR-low group. Genes with no significantly different expression between the two groups are represented by black dots. Shared DEGs identified in both datasets were labeled.

**Figure 7:**
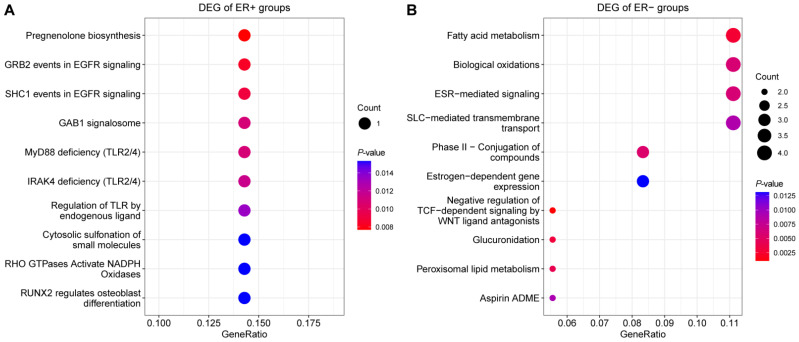
Pathway enrichment of shared DEGs between AR-high and AR-low groups. (**A**) dot plot of the top 10 enriched pathways of the 6 shared DEGs identified in ER+ breast cancer. (**B**) dot plot of the top 10 enriched pathways of the 55 shared DEGs identified in ER− breast cancer.

To further examine the molecular features related to AR-expression in ER-positive and ER-negative breast cancers, we analyzed the deferentially expressed genes (DEGs) between breast cancers with high and low AR expression in TCGA and Molecular Taxonomy of Breast Cancer International Consortium (METABRIC) datasets, respectively. The number of included breast cancer samples in the TCGA and METABRIC datasets are 466 and 1904, respectively. The breast cancer samples were divided into AR-high and AR-low groups using the expression value of the *AR* gene. Breast cancer cases with AR expression in the upper tertile were included in the AR-high group and those in the lower tertile were included in the AR-low group. DEGs between AR-high and AR-low groups in ER-positive breast cancer were analyzed in TCGA and METABRIC datasets respectively (Figure 6A,B). The number of DEGs was higher in the TCGA dataset than in the METABRIC dataset. Six genes were identified as shared DEGs in the two datasets and were labeled in Figure 6A,B and listed in Table 3. To further examine the functions of those DEGs, pathway enrichment analysis of the six shared DEGs was performed using ReactomePA (Figure 7A). The enriched pathways include steroid hormone synthesis and EGFR activation pathway. DEGs between AR-high and AR-low groups in ER-negative breast cancer were also identified in TCGA and METABRIC datasets(Figure 6C,D). There is a total of 55 shared DEGs between the two datasets with 15 of them in the up-regulated group and 40 of them in the down-regulated group (Table 4). The enriched pathway of the 55 shared DEGs are mainly pathways related to cell metabolism and ER regulation (Figure 7B). There is no shared pathway between the ER-positive group and ER-negative group, indicating that molecular mechanisms related to AR expression were different between ER-positive and ER-negative breast cancer cases.

## 3. Discussion

In this study, AR was expressed in 92% and 47% of ER-positive and ER-negative breast cancer cases, respectively, using the PKUCH cohort. Our results show that AR negativity was associated with distinctive clinicopathological characteristics in ER-positive and ER-negative breast cancer. While AR expression only associates with PR positivity in ER-positive breast cancer, it correlates with a greater age at diagnosis and a less advanced histologic grade in ER-negative breast cancer. Our findings differ from the previous study in which AR expression was found to be associated with a lower histologic grade only in ER-positive breast cancer but not in ER-negative breast cancer [24]. In PAM50 defined intrinsic subtypes, the expression of AR in basal-like subtype was less than in luminal A and luminal B subtypes which is consistent with previous studies [7,25,26]. Additionally, the correlation between the expression of *AR* and *ESR1* was only significant in luminal A and basal-like subtype but not in luminal B and HER2-enriched subtypes. As both luminal B and HER2-enriched subtypes are characterized by HER2 expression, it is possible that the correlation between AR and *ESR1* is disrupted by HER2 expression. Moreover, our result showed that the DEGs associated with AR expression is distinct in ER-positive and ER-negative breast cancer, indicating that the molecular mechanisms related to AR expression were different between ER-positive and ER-negative breast cancer cases.

The molecular subtyping of TNBC identified that about 15% to 20% of TNBC cases can be classified as the luminal androgen receptor (LAR) subtype, which has a greater age at diagnosis and low proliferation index [27,28,29]. However, for AR-positive TNBC, the report on the age at diagnosis compared with other TNBC cases is controversial [4,30,31,32,33,34]. Moreover, a previous study has identified no difference between ER−/AR+ and ER−/AR− breast cancer in terms of age at diagnosis [24]. Our result show that ER−/AR+ breast cancer shares similar characteristics with LAR in terms of low proliferation index and older age at diagnosis. Additionally, we found that the expression of AR was correlated with different molecular features in ER-positive and ER-negative breast cancer, suggesting that the role of AR in ER-positive breast cancer may be distinct from that in ER-negative breast cancer.

There are several limitations to our study. First, the PKUCH cohort was a retrospective study without survival information; thus, the prognostic value of AR negativity in ER-positive and ER-negative breast cancer cannot be investigated. Second, our analysis only focused on the HER2-negative breast cancer, and previous studies demonstrated that the correlation between AR pathway activity and AR expression varied in HER2-positive and HER2-negative breast cancer [17,35]. Indeed, studies have shown that breast cancer in the Asian population has different clinicopathology from other populations [36,37]. Moreover, due to the retrospective nature of this study, there is a lack of information about potential confounding factors that may affect our results including smoking [38], body mass index [39] and reproductive history [40].

## 4. Materials and Methods

### 4.1. Study Design

**Figure 8:**
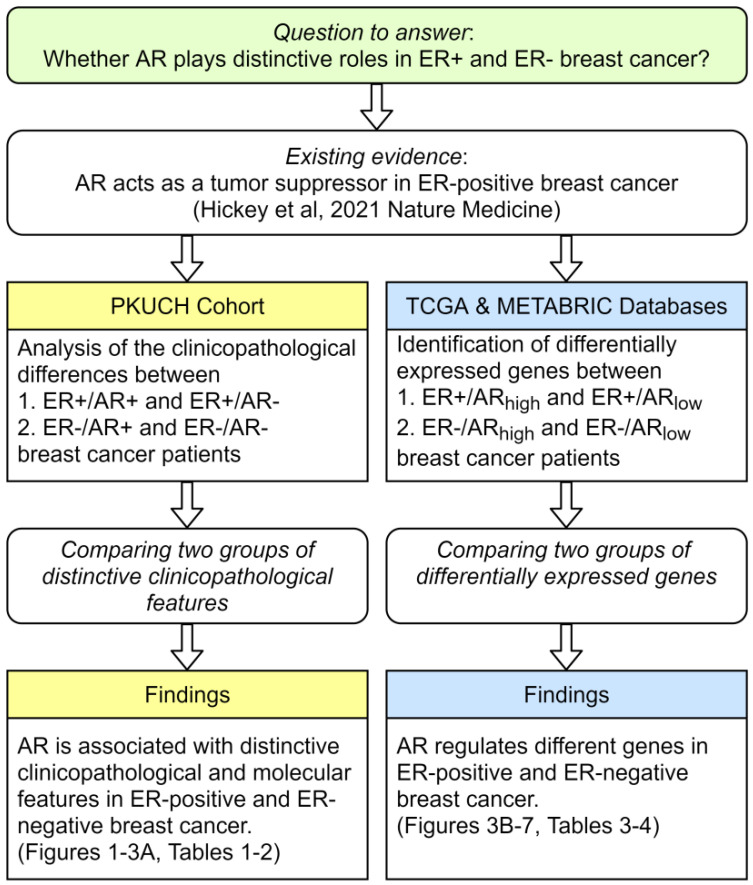
Study design [15].

To answer whether AR plays distinctive roles in ER-positive and ER-negative breast cancer, we analyzed breast cancer cases from three data sources, including the PKUCH cohort, the TCGA dataset and the METABRIC dataset (Figure 8). Using the PKUCH cohort, we analyzed the clinicopathological differences between AR-positive and AR-negative breast cancer in both ER-positive and ER-negative groups. It was found that AR was associated with distinctive clinicopathological and molecular features in ER-positive and ER-negative breast cancer. Meanwhile, we analyzed the differentially expressed genes (DEGs) between AR-positive and AR-negative breast cancer in both ER-positive and ER-negative groups using the TCGA and METABRIC databases, in order to examine whether AR regulated different genes in ER-positive and ER-negative breast cancer.

### 4.2. Patient Selection in PKUCH Cohort

The pathology database in the Peking University Cancer Hospital was queried, and all breast core needle biopsy (CNB) cases diagnosed from September 2019 to May 2021 were retrieved. Selection criteria include a diagnosis of primary invasive breast carcinoma, patient older than 18 years of age, and intact immunostaining information of AR, ER, PR, HER2 and Ki67. A total of 323 breast carcinoma cases were selected. The cases were reviewed by two experienced pathologists (Y Liu and M Long).

### 4.3. Immunohistochemical Staining and Analysis of PKUCH Cohort

Immunohistochemistry of the formalin-fixed, paraffin-embedded tissue was performed as described in our previous study. Antibodies used in this study include ER (SP1; Roche, Tucson, AZ, USA; 1 μg/mL), PR (1E2; Roche, Tucson, AZ, USA; 1 μg/mL), HER2 (4B5; Ventanne, Tucson, AZ, USA; 6 μg/mL) and Ki67 (M1B1; Zhongshanjinqiao, Beijing, China; working concentration). The immunohistochemical stains were evaluated by two pathologists with consensus (M Long and Y Liu). The Ki67 score is defined as the percentage of positively nuclear-stained cells divided by the total number of malignant cells scored. When the staining is homogenous across the sample, a global Ki67 score was used, and for heterogeneous staining, the Ki67 score was counted in the hotspot regions. Positivity of ER, PR and AR was defined as ≥1% of cancer cells showing positive nuclear staining. A comparison of clinicopathological characteristics between two groups was performed using the “summary” function in the “finalfit” package with R (version 4.1.2). Continuous data are compared with a Kruskal-Wallis test, while discrete data are compared with a chi-squared test.

### 4.4. Analysis of Differentially Expressed Genes in Breast Cancer from TCGA

For breast cancer from TCGA [41], RNA-seq and clinical data of 1102 breast cancer samples were downloaded and processed using the TCGAbioloinks R/Bioconductor package (version 2.18.0) [42], as described in a series of our previous publications [43,44,45,46]. The Fragments Per Kilobase of transcript per Million mapped reads Upper Quartile (FPKM-UQ) RNA-seq data were log2-transformed before being further processed. The FPKM-UQ is implemented at the GDC on gene-level read counts that are produced by HTSeq and based on a modified version of the FPKM normalization method [47]. The log2-transformed FPKM-UQ data were analyzed using limma package (version 3.44.3) functions lmFit, eBayes, and topTable to identify DEGs [48]. The threshold for DEGs identification is setted as false discovery rate (FDR) < 0.01 and |log2fold change| > 1.

### 4.5. Analysis of Differentially Expressed Genes in Breast Cancer from METABRIC

The mRNA expression and clinical data of 1904 breast cancer samples from METABRIC study were downloaded via cBioPortal [49,50,51]. For DEGs analysis, the expression log intensity levels generated by Illumina Human v3 microarray were used. Pathway enrichment analysis was performed using ReactomePA package (version 1.40.0) [52].

## 5. Conclusions

Our findings demonstrate that the expression of AR is associated with distinctive clinicopathological and molecular features in ER-positive and ER-negative breast cancer. Further characterization of the altered pathways and molecular features associated with AR expression in both ER-positive and ER-negative breast cancer is required for the precise application of AR-targeted therapy.

## Figures and Tables

**Table 1:** Clinicopathological characteristics of the ER+/AR− and ER+/AR+ groups.

Group	ER+/AR− (*n* = 21)	ER+/AR+ (*n* = 253)	*p*
Age			
Mean (SD)	52.2 (11.7)	53.5 (11.4)	0.603
Grade			
I	1 (4.8)	26 (10.3)	0.062
II	16 (76.2)	212 (83.8)	
III	4 (19.0)	15 (5.9)	
Histology			
IDC-NST	21 (100.0)	243 (96.0)	0.835
Invasive lobular carcinoma	0 (0)	5 (2.0)	
Micropapillary carcinoma	0 (0)	3 (1.2)	
Mucinous carcinoma	0 (0)	2 (0.8)	
EGFR			
Negative	16 (76.2)	210 (83.0)	0.386
Positive	3 (14.3)	16 (6.3)	
Unknown	2 (9.5)	27 (10.7)	
CK5/6			
Negative	19 (90.5)	246 (97.2)	0.168
Positive	2 (9.5)	6 (2.4)	
Unknown	0 (0)	1 (0.4)	
PR			
Negative	5 (23.8)	15 (5.9)	0.010
Positive	16 (76.2)	238 (94.1)	

**Table 2:** Clinicopathological characteristics of the ER−/AR− and ER−/AR+ groups.

Group	ER−/AR− (*n* = 26)	ER−/AR+ (*n* = 23)	*p*
Age			
Mean (SD)	49.1 (12.9)	58.1 (12.9)	0.019
Grade			
I	0 (0)	0 (0)	0.016
II	5 (19.2)	13 (56.5)	
III	21 (80.8)	10 (43.5)	
Histology			
IDC	26 (100.0)	22 (95.7)	0.951
Apocrine Carcinoma	0 (0)	1 (4.3)	
EGFR			
Negative	3 (11.5)	2 (8.7)	0.943
Positive	21 (80.8)	19 (82.6)	
Unknown	2 (7.7)	2 (8.7)	
Ki67			
Mean (SD)	74.0 (12.0)	55.4 (24.5)	0.001
CK5/6			
Negative	6 (23.1)	8 (34.8)	0.556
Positive	20 (76.9)	15 (65.2)	
PR			
Negative	26 (100.0)	19 (82.6)	0.090
Positive	0 (0)	4 (17.4)	

**Table 3:** List of shared differentially expressed genes between ER+/AR-high and ER+/AR-low groups in TCGA and METABRIC databases.

Name	TCGA		METABRIC	
	logFC	FDR	logFC	FDR
*AR*	−1.817124266	2.48 × 10^−51^	−1.759114522	0
*SEC14L2*	−1.456398856	4.22 × 10^−18^	−1.064295136	1.51 × 10^−45^
*SCUBE2*	−1.247527072	6.05 × 10^−8^	−1.160267382	2.79 × 10^−22^
*PIP*	−2.242869262	2.14 × 10^−7^	−1.359740461	2.97 × 10^−13^
*ANKRD30A*	−2.441016285	5.80 × 10^−7^	−1.406985552	6.46 × 10^−18^
*S100A9*	1.105746351	3.28 × 10^−5^	1.042302941	1.32 × 10^−16^

**Table 4:** List of shared differentially expressed genes between ER−/AR-high and ER−/AR-low groups in TCGA and METABRIC databases.

Name	TCGA		METABRIC	
	logFC	FDR	logFC	FDR
*AR*	−4.295925031	1.45 × 10^−27^	−1.901442266	5.66 × 10^−50^
*FOXA1*	−3.213843492	5.08 × 10^−8^	−3.115137129	7.19 × 10^−30^
*NOSTRIN*	−1.40887953	5.49 × 10^−8^	−1.17526932	2.44 × 10^−19^
*HMGCS2*	−5.982850557	1.02 × 10^−7^	−2.142998338	8.17 × 10^−15^
*ANKRD30A*	−5.84612794	2.76 × 10^−7^	−2.019088917	4.89 × 10^−12^
*GATA3*	−2.12904346	3.03 × 10^−7^	−1.015513328	3.14 × 10^−10^
*MLPH*	−2.443517722	4.28 × 10^−7^	−2.48627378	8.21 × 10^−22^
*CYP4Z2P*	−5.757726105	7.55 × 10^−7^	−1.349071614	9.92 × 10^−16^
*RGMA*	1.523559476	7.55 × 10^−7^	1.044105908	3.13 × 10^−11^
*SCUBE2*	−2.061767467	1.15 × 10^−6^	−1.067324511	6.66 × 10^−14^
*TFAP2B*	−4.414408204	2.45 × 10^−6^	−2.248879556	4.39 × 10^−27^
*PIP*	−5.159250207	5.79 × 10^−6^	−3.564551947	4.33 × 10^−18^
*SPDEF*	−2.849330312	6.05 × 10^−6^	−2.786823851	3.59 × 10^−29^
*GABRP*	3.309171931	7.85 × 10^−6^	3.2837105	2.69 × 10^−20^
*CYB5A*	−1.146286537	8.90 × 10^−6^	−1.38527235	4.09 × 10^−16^
*SFRP2*	−1.264969291	1.03 × 10^−5^	−1.216259253	1.80 × 10^−08^
*CNTNAP2*	−2.963699101	1.30 × 10^−5^	−1.31705986	1.65 × 10^−10^
*UGT2B11*	−5.754824596	1.63 × 10^−5^	−2.499376453	5.35 × 10^−13^
*SLC40A1*	−1.57457122	1.66 × 10^−5^	−1.096405114	2.81 × 10^−12^
*C1orf64*	−4.891014419	2.04 × 10^−5^	−1.358795191	3.39 × 10^−10^
*ALCAM*	−1.421797594	3.11 × 10^−5^	−1.492164324	6.29 × 10^−16^
*REEP6*	−2.131080407	3.45 × 10^−5^	−1.991369244	1.26 × 10^−17^
*C9orf152*	−2.474497544	3.89 × 10^−5^	−2.048343549	1.17 × 10^−19^
*TFF3*	−3.56815467	6.54 × 10^−5^	−3.132898878	1.12 × 10^−19^
*CAPN13*	−2.994917078	6.98 × 10^−5^	−1.827529993	1.03 × 10^−16^
*ART3*	3.077955742	9.58 × 10^−5^	2.120528214	2.32 × 10^−14^
*CLCA2*	−3.498079139	9.58 × 10^−5^	−2.085823499	1.91 × 10^−14^
*ABCC11*	−2.825338538	9.66 × 10^−5^	−1.680069132	4.05 × 10^−13^
*FZD9*	1.975719541	9.73 × 10^−5^	1.396728979	1.02 × 10^−15^
*ACOX2*	−1.470170247	0.000111929	−1.023673661	5.08 × 10^−9^
*ROPN1B*	2.719861187	0.000142	2.245409862	1.17 × 10^−19^
*SLC7A2*	−1.595639475	0.000225608	−1.259892508	1.42 × 10^−14^
*SLC44A4*	−2.297987517	0.000261259	−1.232436236	4.19 × 10^−12^
*SHC4*	2.112277227	0.000286784	1.413138021	4.05 × 10^−14^
*SOX8*	2.394006019	0.00030181	1.308657841	1.87 × 10^−07^
*LEMD1*	2.268752567	0.000338694	1.336755062	8.27 × 10^−17^
*MIA*	3.004452058	0.000374416	1.152236253	2.45 × 10^−10^
*SPINK8*	−4.801533789	0.000377021	−1.932031705	3.75 × 10^−16^
*HPGD*	−2.052376193	0.000407842	−1.087467101	1.56 × 10^−06^
*FOXC1*	1.355691626	0.000455715	2.177507604	9.92 × 10^−16^
*LRRC26*	−3.185218295	0.000464176	−2.393564467	8.31 × 10^−20^
*HRCT1*	1.836318744	0.000547387	1.217364186	4.05 × 10^−15^
*SFRP1*	1.703174242	0.000558313	1.892525614	6.54 × 10^−12^
*CYP4Z1*	−3.331023389	0.001049392	−1.898760109	3.49 × 10^−17^
*SLC26A3*	−2.886229901	0.001135615	−1.038268061	1.64 × 10^−7^
*CRAT*	−1.081040223	0.001213952	−1.095934205	1.82 × 10^−15^
*MFGE8*	1.120102102	0.001318467	1.051062396	1.72 × 10^−9^
*CILP*	−1.432888244	0.001543574	−1.047884501	1.77 × 10^−6^
*THRSP*	−2.306889406	0.001957621	−1.202083641	5.36 × 10^−9^
*C2CD4B*	−1.600907683	0.00275741	−1.177058074	5.67 × 10^−11^
*CMBL*	−1.157470421	0.003567629	−1.417268369	2.20 × 10^−13^
*SCGB2A2*	−3.739630635	0.00390212	−2.033977043	3.77 × 10^−6^
*VGLL1*	2.55783435	0.003989974	1.665891121	5.86 × 10^−14^
*SFRP4*	−1.069605879	0.007015003	−1.206349248	1.73 × 10^−8^
*KRT6B*	1.985907376	0.007554538	2.171033331	6.20 × 10^−10^

## Data Availability

All data generated or analyzed during this study are included in this published article.
